# Health Risk Assessment of Metals via Multi-Source Oral Exposure for Children Living in Areas with Intense Electronic Manufacturing Activities

**DOI:** 10.3390/ijerph182111409

**Published:** 2021-10-29

**Authors:** Beibei Wang, Chunye Lin, Hongguang Cheng, Xiaoli Duan, Qin Wang, Dongqun Xu

**Affiliations:** 1 School of Energy and Environmental Engineering, University of Science and Technology Beijing, Beijing 100083, China; wangbeibei723@ustb.edu.cn; 2 State Key Joint Laboratory of Environmental Simulation and Pollution Control, School of Environment, Beijing Normal University, Beijing 100875, China; c.lin@bnu.edu.cn (C.L.); chg@bnu.edu.cn (H.C.); 3 Chinese Center for Disease Control and Prevention, Institute of Environmental Health and Related Product Safety, Beijing 100021, China; wangqin@nieh.chinacdc.cn (Q.W.); xudq@chinacdc.cn (D.X.)

**Keywords:** health risk, children, multi-source ingestion exposure, metal(loid)s, electronic manufacturing activities

## Abstract

Oral ingestion is the predominant pathway of metal(loid)s exposure. In this study, the health risks of typical metal(loid)s (including Mn, As, Cr, Cd, and Pb) via multi-source, oral pathways for children aged 3–12 years, living in an area of China dominated by the electronic manufacturing industry, were studied based on the field sampling of duplicated diet, soil, and drinking water. Child-specific ingestion parameters were measured (except the soil ingestion rates, which were from a previous study of the same population), and a Monte Carlo method was applied to determine the uncertainty of the risk assessment. It was observed that children living in such environments were at risk of metal(loid)s exposure, with the accumulative carcinogenic risk exceeding the maximum acceptable level. Food intake was identified to be the primary exposure pathway. Moreover, Pb and Cr were the major risk elements to local children’s health. Compared with primary school students, kindergarten children experienced a higher risk. This study highlights that high attention should be paid to children living in suburban areas dominated by the electronic manufacturing industry, and that priority should be given to studies on metal(loid)s exposure deriving from different types of food and their corresponding bioavailability, in order to further discern the precise risk sources to protect children’s health.

## 1. Introduction

The chronic exposure to toxic metal(loid)s, such as manganese (Mn), lead (Pb), chromium (Cr), cadmium (Cd) and arsenic (As), is proven to be associated with numerous detrimental health outcomes, including cancer, nervous system disease, visceral organ damage, cardiovascular disease, endocrine disorder, skin damage, and so on [1,2,3,4]. Besides blood, Pb can accumulate in the human skeleton, and move to various target organ systems, such as the kidneys and the brain. The Pb mobilization in bone is associated with the development of several chronic diseases, such as blood pressure, cardiovascular disease, and renal disease [5]. Additionally, more notably, due to the undeveloped immune system and unique activity patterns, such as frequent hand-to-mouth and object-to-mouth behavior, children are much more susceptible to metal(loid)s exposure [6]. 

The health risks resulting from metal(loid)s exposure have attracted continuous attention in recent years [7,8,9]. With the third scientific and technological revolution, the electronic manufacturing industry has developed rapidly all over the world. Meanwhile, it discharges large amounts of toxic chemicals (such as heavy metals), which can enter into soil, water and food through the deposition of ambient particles, surface runoff, and bioaccumulation, posing health hazards to human beings [10,11]. Although studies on the health risks posed by metal(loid)s exposure are well documented, most of them focused on people living in the vicinity of a coking plant, lead-acid battery plant, metal smelter plant, and a solid waste incinerator, among others [12,13,14,15]. However, the possible health risks through the multi-route and element-specific exposure of toxic metals to children, who live in environments polluted by the electronic manufacturing industry, are still not well understood. 

Humans can be exposed to metal(loid)s via several routes: inhalation, food ingestion, and the dermal contact with soil and water. For most metal(loid)s, dietary ingestion is demonstrated to be the dominant exposure pathway in a number of studies [16,17], whereas soil ingestion is another important exposure pathway for some metal(loid)s. For instance, the exposure dose through this pathway strongly affected children’s blood, lead, and urine Cr levels [18,19,20]. In addition, drinking water could also pose adverse health effects to human beings in some areas [21,22]. Therefore, assessing the accumulative oral exposure level could reflect the comprehensive metal(loid)s exposure level to a large extent.

In addition, the exposure parameters play a critical role in health risk assessment, with their accuracy directly determining the precision of the results. For example, the food intake rate contributed more than 50% to the total variance in the cancer risk assessment associated with dietary PAHs exposure [23]. However, previous studies generally use the parameters recommend by other countries during risk calculating. In this situation, due to the discrepancies in behavior patterns among the population [24], a bias might be caused. Therefore, it is necessary to use the subjects’ specific exposure parameters. Shenzhen is one of the most important bases for the electronic manufacturing industry in China. Therefore, the objective of this study was to assess the pollution levels and health risks from the oral exposure of metal(loid)s to children living in the suburban area of Shenzhen. The typical toxic metal(loid)s, including Mn, Pb, Cr, Cd, and As, were measured in duplicates of diet, drinking water, and soil samples, which the children were exposed to daily. Child-specific exposure factors were investigated, excluding the parameter of soil ingestion rates which were obtained using the mass balance tacer method, as in a previous study for the same group children [25]. The oral exposure level and contributions from each environmental medium were estimated to identify the dominant exposure pathway. The noncarcinogenic and carcinogenic risks posed to children by metal(loid)s were assessed, respectively. Additionally, a Monte Carlo simulation, using the probability distributions of each parameter, was conducted to determine the uncertainty of the risk assessment. The result of this study could serve as a basis for the precise and scientific risk control of local children.

## 2. Materials and Methods

### 2.1. Study Site

Shenzhen, where electronic manufacturing is the dominant industry, was featured in this study. Additionally, as the window city of China’s reform and opening-up strategy, it experienced a rapid process of industrial development and urban expansion, with the GDP in 2019 exceeding 2.6 trillion yuan and the population density ranked as fifth in the word at the beginning of 2010, according to Forbes magazine. The vehicle population of Shenzhen reached 3.5 million, ranking first in China with a density of more than 510 cars per kilometer. Moreover, it predominantly has a subtropical marine climate, with a perennial average temperature of 22.5 °C.

### 2.2. Field Sampling and Analysis

#### 2.2.1. Study Design and Population Recruitment

This study was approved by the ethics committee of the National Center for Disease Control and Prevention. A total of 60 children living in suburban areas were randomly recruited to participate in this study, including 30 children aged 3 to 6 years from kindergarten, and 30 children aged 7 to 12 years from primary school. After the informed consent was signed by the children and their guardians, a cross-sectional study was then conducted.

A questionnaire survey was conducted through one-to-one, face-to-face interviews with the subjects, with the assistance of their parents or guardians, to obtain the basic information and the specific exposure parameters of the children, such as height, weight, water intake rate, eating habits, and so on. Moreover, soil ingestion rates were specially studied using the mass balance tracer method, as used in a previous study conducted on the same children [25]. Additionally, three types of environmental samples, including drinking water, a duplicated diet sample, and a soil sample were collected to determine the accumulative oral exposure and health risk levels associated with the target metal(loid)s.

#### 2.2.2. Sample Collection

Food samples were collected using the “duplicated plate method”, with the same kinds and quantities of food as those consumed by the participants. One-day duplicated food samples, including breakfast, lunch, supper, and snacks, were taken between Tuesday morning (about 7 a.m.) and Wednesday morning (about 7 a.m.). After being weighted separately, all the food samples for one subject were homogenized evenly with stainless steel blades to form a composite sample.

A total of 60 tap water was collected from each child’s family and 10 water samples were collected from the water cooler at the children’s schools using 1 L acid-washed polyethylene bottles. After adding two drops of 65% concentrated HNO_3_ into the water, the samples were stored at −20 °C until analysis.

Topsoil samples (at a depth of 0 to 20 cm) were collected from places where children generally played (such as green spaces near the children’s homes or schoolyard). In total, 60 soil samples were collected. In each sampling site, a composite sample was obtained by randomly integrating 4 to 5 equal sub-samples from a 10 cm by 10 cm area. 

#### 2.2.3. Sample Pretreatment and Instrumental Analysis

In the laboratory, water samples were filtered with the filter membrane (Whatman No.1, Φ = 0.45 mm) before analysis. Soil samples were air-dried, crushed with a ceramic mortar and a rubber pestle, and then passed through a 0.25 mm sieve. A 0.5 g subsample was digested with concentrated HNO_3_-HF-HClO_4_ using microwave (MARS-5, CEM, North Carolina, USA) heating to analyze the metal(loid)s concentration. A subsample of food (1 g) was digested with concentrated nitric acid and hydrogen peroxide (HNO_3_-H_2_O_2_) in a microwave oven. 

The concentrations of As, Cr, Cd, Pb, and Mn were determined by ICP-MS (Agilent-7500a, Agilent Scientific Technology Ltd., Palo Alto, Santa Clara, CA, USA) under the optimized conditions [26].

#### 2.2.4. Quality Control

Several quality control measures were taken to ensure the accuracy and reliability of the method for metal(loid)s pretreatment and determination. Every digestion batch involved reagent blank samples, spiked samples, and duplicated samples, with each amount accounting for 10% of the total samples. Additionally, a certified reference material was tested every 10 samples during analyzing.

The detection limits for Mn, As, Cr, Cd, and Pb were 2.0 to 20 μg·kg^−1^. The coefficient of variation for parallel measurements was <5.0%, and the recovery rate of the spiked samples was within the range of 85.1% to 113.6%.

### 2.3. Exposure Assessment and Risk Characterization

According to the exposure assessment models recommended by the US Environmental Protection Agency [27], the average daily dose through the ingesting pathway (*ADD*_ingest_, mg·kg^−1^day^−1^) was calculated using the following equation.

(1) $$ADD_{\mathrm{ingest}}=\frac{C\times I\mathrm{ngR}\times EF\times ED}{BW\times AT}\times 10^{-6}$$

where *C* is the metal(loid) concentration in soil (mg·kg^−1^), food (mg·kg^−1^), or drinking water (μg·L^−1^); *I*ngR is the ingestion rate, whereby soil and food is expressed as mg·day^−1^, and for water is expressed in mL·day^−1^; *EF* is the exposure frequency in day·year^−1^ and the value is 365 days·year^−1^; *ED* is the exposure duration in years; *BW* is the children’s body weight in kg; and *AT* is the average exposure time in days, while for the noncarcinogenic risks, *AT* = *ED* × 365 days. All the exposure factors were present in Table 1. 

A Hazard Quotient (*HQ*) was calculated to assess the non-carcinogenic risk using the following equation [27]:

(2) $$HQ=\frac{ADD}{RfD}$$

where *RfD* is the estimated maximum permissible daily exposure level to humans, without a likely appreciable risk of deleterious effects in mg·kg^−1^day^−1^. The oral *RfD* for Mn, Pb, Cr (diet and soil), Cd (diet and soil), Cd (water), and As were 0.14, 1.5, 0.001, 0.0005, and 0.0003, respectively, which were derived from the Integrated Risk Information System (IRIS) of U.S. EPA based on extensively epidemiological and toxicological data. Additionally, due to the unavailability of IRIS, the oral *RfD* for Pb (0.0014 mg/kg⸱d) and Cr (water) (3 mg/kg⸱d) were the same as the values commonly used in previous studies [14,28,29]. If *HQ* ≤ 1, it is not likely to pose adverse effects on human health. Otherwise, potential health risk could occur. In addition, a Hazard Index (*HI*) was used to assess the cumulative non-carcinogenic risk posed by multiple metal(loid)s and the total Hazard Index (*HIt*) was used to assess the aggregative non-carcinogenic risk through multiple exposure pathways:

(3) $$HI=\sum_{1}^{i}HQ$$

(4) $$HIt=\sum_{1}^{i}HI$$

### 2.4. Uncertainty Analysis

Uncertainty of the estimations of health risk could be derived from any of the parameters, such as the metal(loid) concentration, body weight, and ingestion rate. To determine the uncertainty, a Monte Carlo simulation was employed with Crystal Ball software (16.0) (ORACLE, Beijing, China) using 10,000 iterations to manage the variabilities both in metal(loid) concentrations and in parameters.

### 2.5. Statistical Analysis

The content of metal(loid)s in each environmental medium and the risk level through each route were presented as median and percentile values. The correlation coefficients were calculated using the Spearman’s method. The Kolmogorov–Smirnov test was used to identify whether the data were normally distributed, and Mann–Whitney U test was used to compare the means when the data were not normally distributed. The whole statistical analysis was conducted by SPSS 20.0 (IBM, New York, USA) with a significance level of 0.05 for two-tailed testing.

## 3. Results and Discussion

### 3.1. Metal(Loid)s in Environment Media

#### 3.1.1. Duplicated Diet 

The concentrations of metal(loid)s in food decreased in the following order: Mn > Cr > Pb > As > Cd (Table 2). The metal(loid)s levels determined in the current study were within the range of those reported in the fifth China total diet study, conducted in Guangdong province (Pb: 0.0001–0.33 mg·kg^−1^, Cr: 0–0.45 mg·kg^−1^, Mn: 0.04–11.09 mg·kg^−1^, Cd: 0.0005–2.599 mg·kg^−1^, and As: 0–1.4 mg·kg^−1^) [10], but higher than those reported in the second French total diet study (Cd: 0.0008–0.167 mg·kg^−1^; Pb: 0.023–0.113 mg·kg^−1^) [30] and a dietary exposure study in Spain (Cd: 0.001–0.117 mg·kg^−1^; Pb: 0.002–0.045 mg·kg^−1^) [31], in which a wide range of food types were involved. As found in previous studies, due to the discrepancy in the bioaccumulation capacity and the dependent soil characteristics, metal(loid)s levels varied greatly among food types [32]. The duplicated plate method, which collected a mixture of food consumed throughout the day, combined the difference in metal(loid)s distribution and dietary patterns across the food types. In addition, the influence caused by food processing can also be taken into account.

In comparison with the metal(loid)s levels in the duplicate diets, Pb and Cr concentrations were much higher than those originating from the market (Pb: 0.009 mg·kg^−1^, Cr: 0.022 mg·kg^−1^), as reported in a pilot study [6]. However, compared with the duplicate diet mainly containing locally grown products affected by intense industrial activities, such as intense nonferrous metal mining plants (Pb: 0.49 mg·kg^−1^, Cr: 0.26 mg·kg^−1^) [13] and lead-acid battery plants (Pb: 0.52 mg·kg^−1^, Cr: 0.45 mg·kg^−1^) [15], metal(loid)s levels were much lower but almost in the same order of magnitude. The enrichment of Pb and Cr in food was largely attributable to electronic manufacturing activities, with Pb and Cr the dominant contaminants [11,33]. 

The age differences between children with metal(loid)s contents in their diets were further explored; the results showed that Pb and Cr in the diets of children aged 3–6 years were significantly higher than in children aged 7–12 years (*p* < 0.05). Meat was identified as the food containing higher levels of Pb and Cr compared with the other food types [34,35]. Meanwhile, according to the first Chinese Environmental Exposure-Related Human Activity Patterns Survey-Children (CEERHAPS-C), younger children had a higher proportion of meat intake than older children [24]. Therefore, the age difference in children affected by Pb and Cr might be largely explained by the discrepancy in the enrichment capacity of metal(loid)s across the food types and the differences in diet structure between the age groups.

In general, most metal(loid)s in food showed no significant correlations, indicating the complexity of the pollution source. For example, atmospheric deposition was proved to be the major source of Pb in leafy vegetables [36], while Cd and Pb may originate from the application of compound fertilizers and pesticides [37]. In addition, no significant correlations were observed between the metal(loid)s in food and in soil, which was in consistent with the previous studies [6,38]. This could be largely explained by the fact that the soil sampled in this study was not farmland soil, although most of the food consumed was planted in the study area, which might be affected by the local pollution.

#### 3.1.2. Soil

The distribution of the target metal(loid)s concentration in soil is presented in Table 2, and the comparison with the background values and those from other studies are displayed in Table 3. The ranking of the metal(loid)s concentration was the same as that in food. Based on the latest soil environmental quality standard of China (GB 36600-2018), the average concentrations of those metal(loid)s in soil were all below the relative risk screening value regulated for the development land. However, compared with the average soil background values in Guangdong province [39], the median concentrations for Cd, Pb, Cr, and Mn were about 4.5, 2.0, 1.5 and 1.2 times greater than the corresponding values, respectively. Additionally, approximately 88% of Cd samples, 93% of Pb samples, 57% of Cr samples, and 82% of Mn samples surpassed the background values, indicating the accumulation of metal(loid)s by human activities to varying degrees.

The Pb and Cr levels in this study were comparable to or higher than those previously reported in non-agricultural soil influenced by mining (Pb 48 mg·kg^−1^; Cr 21.7 mg·kg^−1^) [40], coking (Cr 81.51 mg·kg^−1^; Pb 24.10 mg·kg^−1^) [14], and electronic waste processing activities (Cr 57.47 mg·kg^−1^) [41]. Additionally, compared with the mean values reported in the Chinese national soil pollution survey [33], the Pb and Cr levels measured in this study were much greater. This was especially true for Pb, which was nearly twice the national average level. The enrichment of Pb and Cr could be largely explained as the study area was dominated by electronics manufacturing facilities and Pb and Cr were identified to be the dominant characteristic pollutants [11,42,43]. Moreover, the vehicle density in the study area was heavy and traffic-related activities were proven to be an important pollution source of Pb [44], so the traffic source somewhat contributed to the high concentration of Pb in soil.

Strong correlations were observed between most of the metal(loid)s in soil. For instance, As was significantly positively correlated with Cr (Spearman r = 0.499, *p* < 0.01) and Cd showed significant positive correlations with Mn (Spearman r = 0.466, *p* < 0.01), Pb (Spearman r = 0.484, *p* < 0.01), and Cr (Spearman r = 0.405, *p* < 0.01), respectively, implying the similar pollution source.

#### 3.1.3. Drinking Water

Tap water in the present study was considered to be safe for humans as the total metal(loid)s concentration was less than the threshold value regulated in the National Drinking Water Quality Standard (GB5749-2006). However, consistent with the study conducted by Zhang [38], a large variance was found in several metal(loid)s, such as Cr and Cd, with the coefficient of variance more than 100%, implying that the secondary pollution source of drinking water might be present.

### 3.2. Daily Exposure Dose

Human exposure to metal(loid)s could occur via the ingestion of food, drinking water, and soil. Children’s daily exposure doses of metal(loid)s were evaluated based on the concentration levels of metal(loid)s in various environmental mediums and their specific exposure parameters. The intake rates were critical exposure parameters in the oral exposure assessment. Since the duplicated dietary method was used for food sampling, the food ingestion rate was obtained through the actual weighing. The water intake rate was obtained through the means of field questionnaires, while the soil ingestion rates were determined through the mass-balance tracer method specifically conducted in another study for the same children [25]. The exposure parameters of children in different age groups showed a great variation. For example, children aged 7–12 years ingested an average of 66 mg·day^−1^ soil, which was much higher than the 40 mg·day^−1^ for children aged 3–6 years [25].

**Figure 1:**
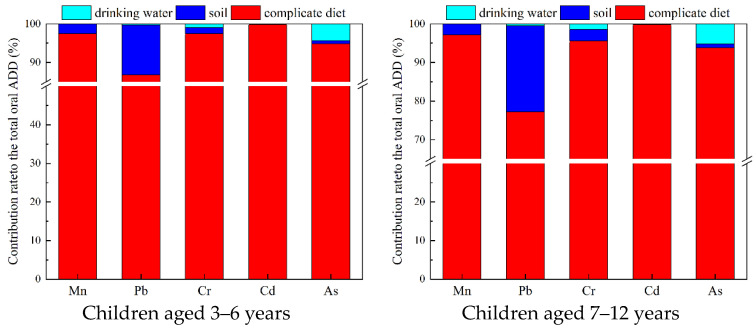
The contribution of exposure to metal(loid)s via the ingestion of food, drinking water, and soil to the total daily oral exposure dose for children (3–6 years and 7–12 years, respectively).

Considering the discrepancy in human activity patterns related to environmental exposure, children were divided into two age groups, 3–6 years old and 7–12 years old, for respective exposure assessments. The contribution of each exposure pathway to the total daily oral exposure dose was shown in Figure 1. Food intake was the dominant exposure pathway for all metal(loid)s, with the contribution rate ranging from 77.2% to 99.7%. In addition, soil ingestion was another important exposure pathway for children aged 7–12 years, with a contribution rate of 22.36%. This result was consistent with a previous study which found strong associations between the neighborhood soil Pb exposure via the ingestion pathway and elevated blood lead levels for urban school-aged children [18], and both of these parameters demonstrated the significance of soil ingestion for school-aged children’s exposure to Pb. 

For most metal(loid)s, the exposure contributions resulting from each environmental medium had similar profiles for children in different age groups. However, the contribution of the soil ingestion pathway to Pb intake for children aged 7–12 years was 1.5 times greater than for children aged 3–6 years. Since there was no significant soil metal(loid)s concentration difference between these two age groups, a possible reason for this was that primary school children ingested more soil than 3–6-year-old children [25]. 

### 3.3. Risk Characterization

The non-carcinogenic risk of oral exposure to various metal(loid)s through multiple sources are shown at the 5th, median, and 95th percentiles in Table 4. The median hazard index for all five metal(loid)s via the ingestion pathways (HIt = 1.9) exceeded the maximum acceptable risk level (1.0), which was comparable to those reported in previous studies for children in the vicinity of the contaminated industry, such as a gold mine in Ghana [12] and a typical lead-acid battery plant in China [15]. This implied that children living in the suburban area dominated by the electronic manufacturing industry may suffer from potential detrimental health effects through exposure to metal(loid)s through ingestion.

**Figure 2:**
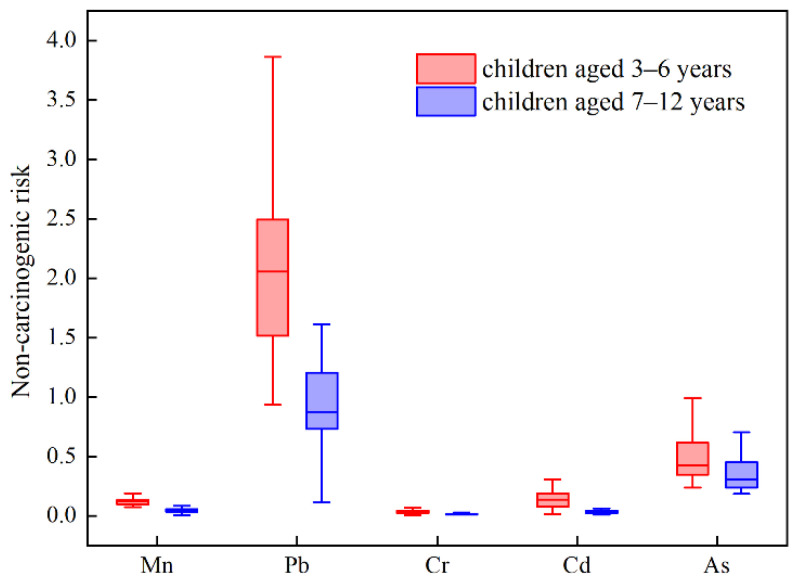
The non-carcinogenic risk of each metal(loid) exposure via ingestion for children aged 3–6 years and 7–12 years, respectively.

Compared with the primary school students, kindergarten children experienced a statistically higher non-carcinogenic risk posed by most metal(loid)s through multi-source ingestion (*p* < 0.05) (Figure 2). This discrepancy was largely due to the higher metal(loid)s content in diet (such as Pb and Cr) and the greater food intake rate per unit of body weight for younger children. In summary, food ingestion was the primary exposure route, accounting for 76.9% of the total risk. Pb was the most high-risk element to local children’s health, contributing approximately 69.6% to the total risk. Furthermore, it is worth noting that the *p* 95 percentile of non-carcinogenic risk, associated with accumulative As ingestion, also exceeded the acceptable level.

### 3.4. Uncertainty Analysis

There are some uncertainties inherent in health risk assessment. First, particular uncertainties existed in the low-dose extrapolation during dose-response assessments, including from animal to human, from the average human to sensitive human, from sub-chronic to long-term exposure, and so on [45]. Second, this study didn’t take into account the bioavailability of each metal(loid) and the total content of the metal(loid)s were applied in risk assessment, and so the risk level of children might be overestimated to some extent. Moreover, a snapshot sampling in the present study may not completely represent longitudinal exposure in the long term. However, several measures were taken to reduce the uncertainty. The exposure parameters applied in the process of risk calculating, especially the soil ingestion rate, were all derived from field surveys and measurements. In addition, the duplicated plate method was also applied for food sampling to avoid the effect on the contamination level deriving from food processing. 

**Figure 3:**
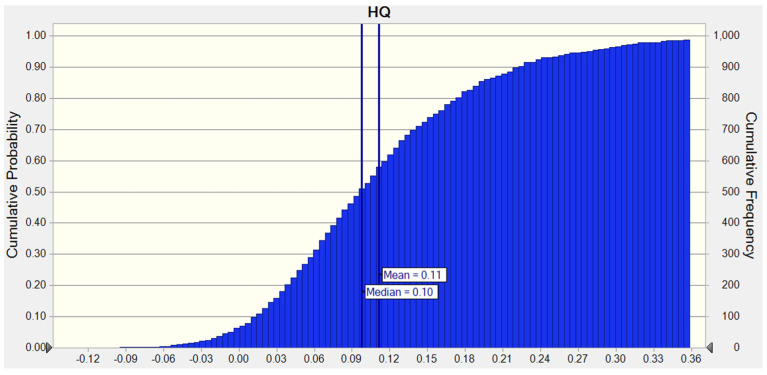
Cumulative probability distribution of the HQ of Pb exposure via soil ingestion pathway.

To assess the uncertainties associated with the calculation process, a Monte Carlo simulation was performed to estimate the cumulative probability distribution of the HQ of Pb exposure through the soil ingestion pathway as an example (Figure 3). It was found that the mean (0.11) and median (0.10) values were close to the calculated value, 9.9 × 10^−2^, indicating that there was likely no bias in the assessment of health risk in our study.

The health risk was evaluated by a Monte Carlo simulation based on Crystal Ball soft for 10,000 iterations.

## 4. Conclusions

This study estimated the potential health risk posed by the oral exposure, for children living in a suburban area dominated by the electronic manufacturing industry, to metal(loid)s. It was observed that children living in such environments were at risk of metal(loid)s exposure, with the aggregated carcinogenic and non-carcinogenic risks all exceeding the maximum acceptable level, which was comparable to the risk levels of those living in typically contaminated areas. Homegrown food intake was the dominant exposure pathway for all the metal(loid)s, while Pb was the most high-risk element.

This study emphasized the importance of protecting children who live in suburban areas dominated by the electronic manufacturing industry. Additionally, a high priority should be given to studies on metal(loid)s exposure resulting from different types of food and their corresponding bioavailability, in order to further identify the precise risk sources.

## Figures and Tables

**Table 1:** Summary of exposure factors developed for the investigation site.

Exposure Parameters	Average		3–6 Years		7–12 Years		Reference
	Median	Mean	Median	Mean	Median	Mean	
Body weight (kg)	25	27	17	19	32	30	Measured
Food ingestion rate (g·day^−1^)	548	545	513	494	612	595	Measured
Soil ingestion rate (mg·day^−1^)	41	51	40	36	66	62	[24]
Water intake rate (mL·day^−1^)	1104	1083	953	937	1238	1254	Measured

**Table 2:** Summary of the concentration of metal(loid)s in various environmental media.

Environmental Media	Sample Size	Value	Mn	Pb	Cr	Cd	As
Soil (mg/kg)	60	median	344.19	72.58	73.65	0.25	7.82
		P25	299.02	56.77	46.21	0.19	4.83
		P75	450.69	81.48	96.04	0.40	12.31
Duplicate diet (mg/kg)	60	median	0.47	0.10	0.13	0.007	0.018
		P25	0.35	0.04	0.08	0.001	0.014
		P75	0.65	0.25	0.18	0.013	0.030
Water (ng/mL)	70	median	0.59	0.13	0.94	0.01	1.41
		P25	0.33	0.07	0.44	0.01	0.89
		P75	1.03	0.22	2.21	0.01	1.78

**Table 3:** Comparison of metal(loid)s concentration in soil in this study with background values and those from other studies (mg/kg).

Mn	Pb	Cr	Cd	As	Reference
344.19	72.58	73.65	0.25	7.82	This study
279.0	36.0	50.5	0.056	8.9	[37]
-	48.0	21.7	0.3	7.9	[38]
593.1	24.1	81.5	0.3	51.5	[13]
-	460.4	57.5	8.2	8.7	[39]

“-” represent no available data.

**Table 4:** Summary of the Hazard Index of the metal(loid)s found in duplicates of diet, water, and soil samples ingested by children at the 5th, median, and 95th percentiles.

	5%				Median				95%			
	Food	Water	Soil	Sum	Food	Water	Soil	Sum	Food	Water	Soil	Sum
Mn	3.1 × 10^−2^	2.1 × 10^−4^	3.0 × 10^−4^	3.1 × 10^−2^	8.1 × 10^−2^	3.4 × 10^−4^	4.9 × 10^−3^	8.6 × 10^−2^	1.7 × 10^−1^	4.6 × 10^−4^	1.5 × 10^−2^	1.8 × 10^−1^
Pb	9.5 × 10^−3^	5.0 × 10^−3^	2.0 × 10^−2^	3.4 × 10^−2^	1.2	8.2 × 10^−3^	9.9 × 10^−2^	1.3	2.9	1.1 × 10^−2^	2.6 × 10^−1^	3.2
Cr	7.2 × 10^−4^	1.6 × 10^−5^	8.8 × 10^−6^	7.5 × 10^−4^	2.1 × 10^−3^	2.7 × 10^−5^	7.8 × 10^−5^	2.2 × 10^−3^	5.9 × 10^−3^	3.6 × 10^−5^	2.6 × 10^−4^	6.2 × 10^−3^
Cd	5.4 × 10^−5^	6.1 × 10^−3^	6.0 × 10^−5^	6.3 × 10^−3^	5.1 × 10^−2^	1.0 × 10^−2^	4.7 × 10^−4^	6.2 × 10^−2^	2.2 × 10^−1^	1.4 × 10^−2^	2.4 × 10^−3^	2.4 × 10^−1^
As	0	1.8 × 10^−1^	3.0 × 10^−3^	1.8 × 10^−1^	5.8 × 10^−2^	2.9 × 10^−1^	3.0 × 10^−2^	3.8 × 10^−1^	5.3 × 10^−1^	4.0 × 10^−1^	1.5 × 10^−1^	1.1
Total	7.0 × 10^−2^	1.9 × 10^−1^	2.3 × 10^−2^	2.8 × 10^−1^	1.5	3.1 × 10^−1^	1.3 × 10^−1^	1.9	3.9	4.2 × 10^−1^	4.3 × 10^−1^	4.8
